# MRI appearance change during stereotactic radiotherapy for large brain metastases and importance of treatment plan modification during treatment period

**DOI:** 10.1007/s11604-019-00886-4

**Published:** 2019-10-15

**Authors:** Katsumaro Kubo, Masahiro Kenjo, Yoshiko Doi, Minoru Nakao, Hideharu Miura, Shuichi Ozawa, Yasushi Nagata

**Affiliations:** 1 Hiroshima High-Precision Radiotherapy Cancer Center, 2-2 Futabanosato Higashi-ku Hiroshima-shi, Hiroshima, 732-0057 Japan; 2 Department of Radiation Oncology, Hiroshima University Hospital, 1-2-3 Kasumi Minami-ku Hiroshima-shi, Hiroshima, 734-8553 Japan

**Keywords:** brain metastasis, magnetic resonance imaging, stereotactic radiotherapy

## Abstract

**Purpose:**

We aimed to evaluate the magnetic resonance imaging (MRI) appearance changes during stereotactic radiotherapy (SRT) for large sized brain metastases, and analyze the lesions necessitating treatment plan modification.

**Materials and methods:**

A total of 23 patients (27 lesions, >2 cm in tumor diameter) underwent SRT and all lesions were evaluated the appearance changes which had the necessity of the treatment plan modification. The appearance change of tumor during SRT was evaluated using gadolinium-enhanced MRI. The reasons of the modification were classified into tumor reduction, tumor enlargement, displacement, and shape change.

**Results:**

Among the 27 lesions, 55.6% required the treatment plan modification. The reasons were tumor reduction in six lesions, tumor enlargement in three lesions, displacement in three lesions, and shape change in three lesions. The planning target volume (PTV) size changed up to 43.0% and the shift of center of PTV was a maximum of 1.7 mm. The pathological status (adenocarcinoma vs others) and timing of steroid administration (prior vs after SRT start) were the predictive factors of tumor changes required the modification.

**Conclusions:**

As tumor changes might occur even during short period of SRT, the treatment plan evaluation and modification were important in SRT for large brain metastases.

## Introduction

Stereotactic irradiation (STI) for brain metastasis, including stereotactic radiosurgery (SRS) and stereotactic radiotherapy (SRT), is a well-established treatment option [1, 2]. SRS administered in a single fraction using gamma knife or linac has been a standard treatment for small brain metastasis, and SRT administered in multiple fractions using gamma knife or linac has been considered for patients with large brain metastasis to reduce the risk of radiation necrosis while providing similar or improved local control [3–8]. In recent times, the demand for STI has increased for its improved local control and prevention of normal brain damage.

On the other hand, in the case of linac-based SRT for large brain metastasis, there is a concern about the tumor changes during the treatment period, such as tumor size, shape, and geometry. With the increase of fraction, the possibility that the tumor changes occur might increase. The changes in tumor size, shape, and geometry during SRT could affect the treatment, such as insufficient target coverage and unnecessary irradiation to normal tissue. However, there were few studies that have assessed the need for treatment plan evaluation and modification during the treatment period of SRT [9, 10], and the specific cases requiring attention remain unclear.

Therefore, this study aimed to evaluate the magnetic resonance imaging (MRI) appearance changes during SRT for large brain metastases, and analyze the lesions necessitating treatment plan modification.

## Materials and methods

From December 2015 to May 2019, patients who met the following criteria were analyzed:

1. Histological or clinical diagnosis of an extracranial primary solid malignant tumor.
2. Patients who received MRI during linac-based SRT to evaluate the necessity of the treatment plan modification.
3. Single or multiple brain metastases with a maximum diameter >2 cm or GTV >5 cc on gadolinium enhanced (Gd) T1-weighted MRI.

Patients who received previous treatment were not excluded. Adjuvant radiation therapy, such as postoperative setting, was not included in this study. The study was approved by the Human Ethics Review Committee of Hiroshima University, and each subject provided written informed consent.

### Treatment

**Fig. 1:**
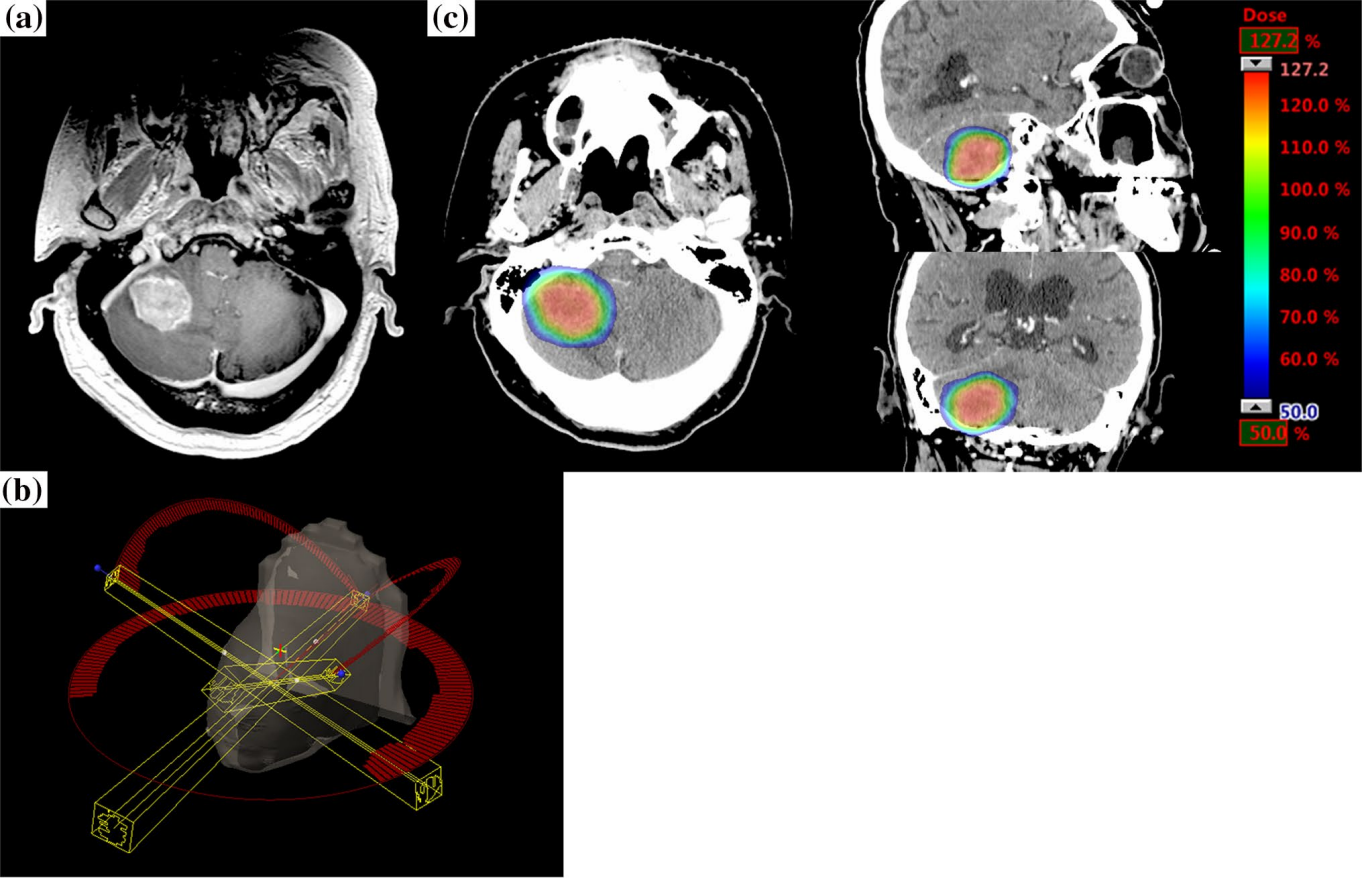
Treatment plan of brain metastasis by volumetric-modulated arc therapy (VMAT).** a** Axial: T1-weighted Magnetic resonance imaging (MRI) with gadolinium (Gd) before stereotactic radiotherapy (SRT). Gross tumor volume (GTV) was defined as the abnormality on the T1-weighted MRI with Gd. Planning target volume was generated by adding a 1-mm margin from GTV. **b** Axial coplanar and two non-coplanar arcs of VMAT. **c** Axial, Sagittal, Coronal: Dose distribution of SRT using VMAT

Before treatment, all patients’ heads were immobilized with non-invasive thermoplastic head mask and were subjected to both contrast-enhanced computed tomography (CT) and Gd MRI scans. The CT and MRI images were acquired with 1.25 mm slice thickness and imported to the iPlan treatment planning system (Version 4.1.2; Brainlab AG, Munich, Germany) or Eclipse treatment planning system (Version 13.5; Varian Medical Systems, Palo Alto, CA, USA) for dynamic conformal arc therapy (DCAT) or volumetric-modulated arc therapy (VMAT) planning (Rapidarc; Varian Medical Systems, Palo Alto, CA, USA). Gross tumor volume (GTV) was defined as the abnormality on the T1-weighted MRI with Gd. Clinical target volume was equal to GTV. Planning target volume (PTV) was generated by adding a 1-mm margin from GTV. The isocenter was located at the center of PTV. Axial coplanar arc of 360 degree and two or three non-coplanar arcs of 180° were used for DCAT and VMAT. Figure 1 shows the treatment planning of VMAT for large brain metastases. All treatment plans were designed based on a TrueBeam STx linear accelerator equipped with 2.5 mm leaf-width multi-leaf collimators (Varian Medical Systems, Palo Alto, CA, USA). The prescribed dose and fraction were chosen based on the size of brain metastasis: for lesions between 2.1 and 3 cm or 5.1 and 10 cc, the total dose was 35 Gy in five fractions, and between 3.1 and 4 cm or 10.1 and 30 cc, the total dose was 40 Gy in eight fractions measured using 6 MV flattening filter-free beams at a maximum dose rate of 1400 MU/min. All treatment were given on consecutive days except weekend days and holiday. The dose was prescribed with the 80% isodose line covering the PTV. Usually, our plans were normalized so that PTV D95 or D98 was equal to the prescribed dose, and GTV D99 (dose that covers 99% of the GTV) was planned to be more than 110% of the prescribed dose. The treatment was started within 60 h of acquiring the treatment planning images. ExacTrac (Version 6.2.1; Brainlab AG, Munich, Germany) was performed daily for patient set up and positioning verification.

During the treatment period, we evaluated the necessity of the treatment plan modification in order to avoid the effect of the changes of tumor size, shape, and geometry. Usually, we conducted Gd-MRI for the evaluation and modification before or after fourth treatment. We decided the modification when the tumor reduction was evident and the modification could reduce high doses to normal brain tissue, or when the tumor was not covered enough by PTV margin and/or the GTV D99 decreased due to tumor enlargement, displacement and shape change. The modified treatment was started within 30 hours of acquiring the MRI images. The patients often received oral dexamethasone of 4.0 mg/day for three consecutive days for preventing cerebral hypertension. Administration of oral dexamethasone was gradually tapered and stopped in about 2 weeks.

### Evaluation and statistical methods

According to the previous report, large brain metastasis was defined as tumor with a maximum diameter of >2 cm [3]. We analyzed the characteristics of large brain metastases with and without the treatment plan modification during the treatment period. The reasons of the modification were classified into tumor reduction, tumor enlargement, displacement, and shape change (Table 1). Tumor reduction was defined as a ≥1 mm reduction in the maximum tumor diameter and ≥10% reduction in the tumor volume on T1-weighted MRI during treatment period. Tumor enlargement was defined as a ≥1 mm enlargement in the tumor diameter in each direction and ≥10% enlargement in the tumor volume on T1-weighted MRI. Displacement was defined as a ≥1 mm shift of center of PTV except tumor reduction and tumor enlargement. Shape change was defined as a slightly change of GTV delineation that caused the decreased GTV D99 except the cases of tumor reduction, tumor enlargement, and displacement. We also evaluated the timing of MRI for the treatment plan modification and the GTV and PTV size change in the cases of tumor reduction and enlargement. In the cases of tumor reduction, we evaluated the brain V70 (volume of normal brain that receives at least 70% of the prescribed dose) before and after the modification to assess the dose to the normal brain. In the all cases, we evaluated the new GTV D99 based on MRI during SRT to assess the dose to the tumor. In the cases of tumor enlargement, displacement, and shape change, the difference of the new GTV D99 of previous plan and that of replan was evaluated by Student’s* t*-test. Univariate analyses using the χ^2^ test were performed to determine the statistical significance of differences between lesions with and those without the modification. These were examined by the reasons of the modification because the factors involved in each reason were expected to be different. Since displacement and shape change were considered to have the same factors affecting them, such as surrounding edema, they were examined together. Investigated factors included the sex, pathological status, location, presence of cystic component on MRI scans, previous treatment, PTV size, dose per fraction, and timing of steroid administration. In this analysis, the patient without pathological diagnosis of primary tumor were excluded. BellCurve for Excel (version 3.20; Social Survey Research Information Co., Ltd., Tokyo, Japan) was used to perform the statistical analyses. Statistical significance was defined as* P* < 0.05.

**Table 1:** Definition of each reason of modification

	Definition
Tumor reduction	≥1 mm reduction in the maximum tumor diameter and ≥10% reduction in the tumor volume
Tumor enlargement	≥1 mm enlargement in the tumor diameter in each direction and ≥10% enlargement in the tumor volume
Displacement	≥1 mm shift of center of PTV except tumor reduction and tumor enlargement
Shape change	A slightly change of GTV delineation that caused the decreased GTV D99 except the above cases

*PTV* planning target volume,* GTV* gross tumor volume,* GTV D99* dose that covers 99% of the GTV

## Results

### Patients

Twenty-three patients with 27 lesions were enrolled in this study. The characteristics of the eligible patients and tumor are summarized in Table 2. The patients’ median age was 68 years (range 46–91 years), and 56.5% of the patients were men. Recursive partitioning analysis (RPA) class I was observed in three patients, and RPA class II was in 20 patients, respectively [11]. Primary tumor sites were lung in 10 patients, breast in 4 patients, gastrointestinal tract in 4 patients, and other in 5 patients. Ten (37.0%) lesions previously received intracranial treatment. Among them, whole brain radiation therapy was administered in six lesions. Median tumor diameter was 2.6 cm (range 2.1–4.0 cm), median tumor volume was 8.1 cc (range 3.2–33.0 cc), and median PTV size was 11.9 cc (range 4.8–39.7 cc). The total prescribed doses were 35 Gy in 5 fractions in 19 lesions and 40 Gy in 8 fractions in 8 lesions. The median GTV D99 with prescribed dose of 35 Gy and 40 Gy were 39.2 Gy (range 36.8–40.3 Gy) and 45.4 Gy (range 44.1–47.4 Gy), respectively. Four lesions were treated by DCAT, and 23 were by VMAT.

**Table 2:** Patient and tumor characteristics

Patients	*N* = 23	100 (%)
Age, years, median (range)	68 (46–91)	-
Sex		
Male	13	56.5
Female	10	43.5
RPA class		
I	3	13.0
II	20	87.0
Primary tumor site		
Lung	10	43.5
Breast	4	17.4
Gastrointestinal tract	4	17.4
Other	5	21.7
Previous therapy (Yes)	8	34.8
Whole brain radiation therapy	4	17.4
Local radiation therapy	3	13.0
SRT	1	4.3
Tumor	*N* = 27	100 (%)
Diameter, cm, median (range)	2.6 (2.1–4.0)	-
GTV size, cc, median (range)	8.1 (3.2–33.0)	-
PTV size, cc, median (range)	11.9 (4.8–39.7)	-
Prescribed dose		
35 Gy/5 fraction	19	70.4
40 Gy/8 fraction	8	29.6
GTV D99, Gy, median (range)		
35 Gy/5 fraction	39.2 (36.8–40.3)	-
40 Gy/8 fraction	45.4 (44.1–47.4)	-
VMAT (Yes)	23	85.2

*RPA* Recursive partitioning analysis,* SRT* stereotactic radiotherapy,* GTV* gross tumor volume,* PTV* planning target volume,* VMAT* volumetric-modulated arc therapy,* GTV D99* dose that covers 99% of the GTV

### Modification of treatment planning

**Fig. 2:**
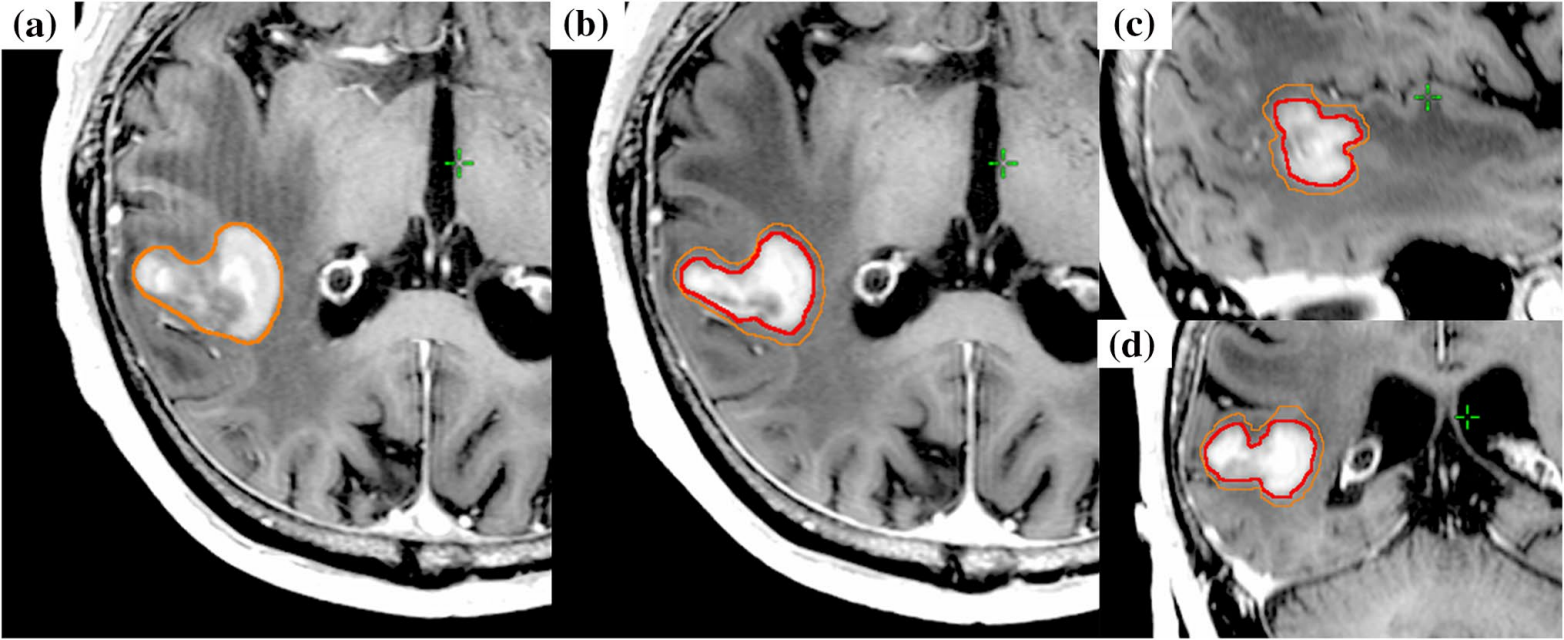
The case of tumor volume reduction during stereotactic radiotherapy. The case was brain metastasis of which primary tumor was colon adenocarcinoma. Orange line shows the gross tumor volume (GTV) of the initial plan, and red line shows GTV at the modification. **a** Axial: T1-weighted magnetic resonance imaging (MRI) with gadolinium (Gd) of the initial plan. **b, c, d** Axial, Sagittal, Coronal: T1-weighted MRI with Gd before 3rd treatment for the modification (the fifth day of SRT start)

**Fig. 3:**
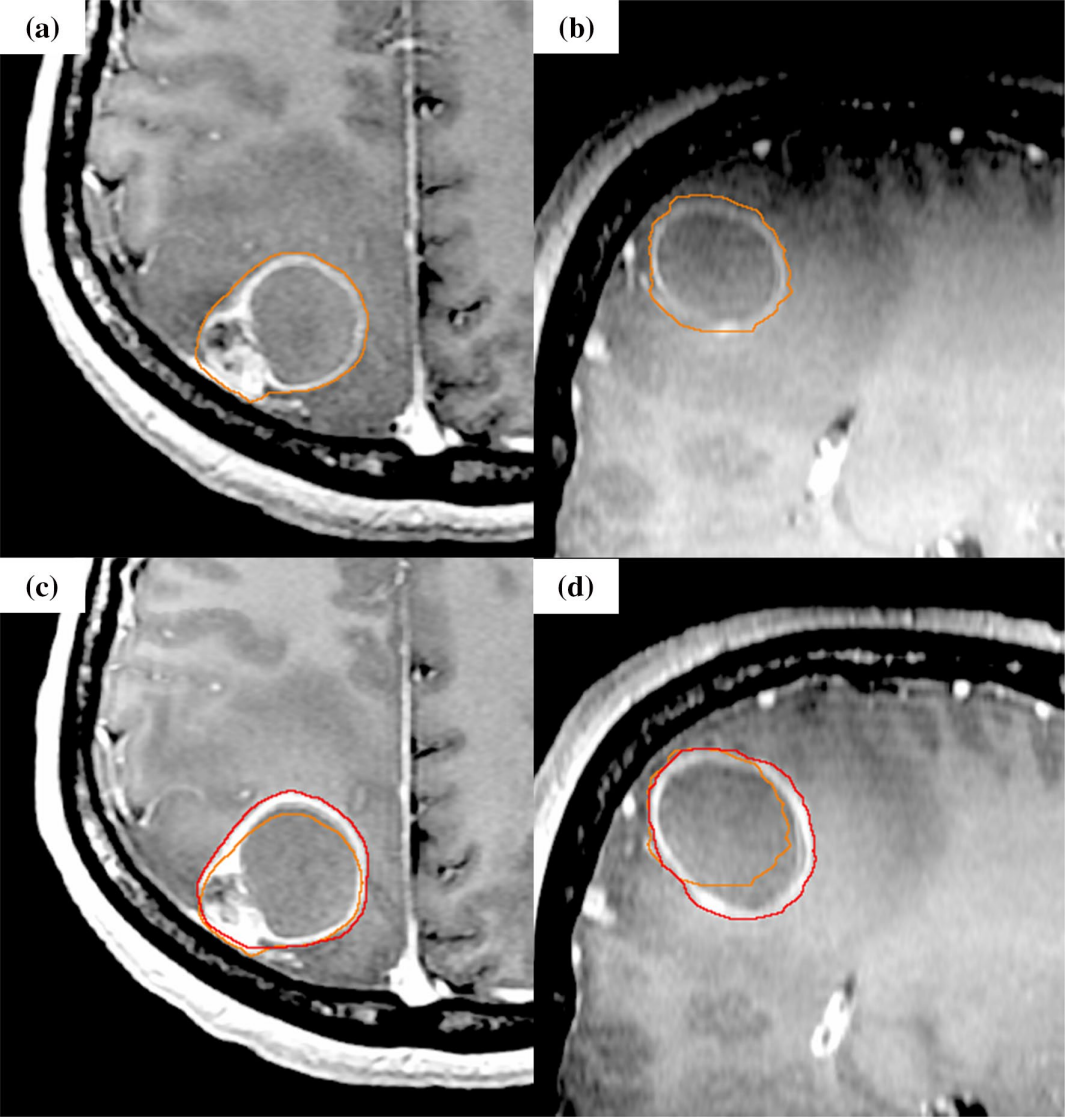
The case of tumor enlargement during stereotactic radiotherapy. The case was brain metastasis of which primary tumor was skin melanoma. Orange line shows the gross tumor volume (GTV) of the initial plan, and red line shows GTV at the modification. The enlargement of cystic component was observed. **a, b** Axial, Sagittal: T1-weighted magnetic resonance imaging (MRI) with gadolinium (Gd) of the initial plan.** c, d** Axial, Sagittal: T1-weighted MRI with Gd before fourth treatment for the modification (the 6th day of SRT start)

**Fig. 4:**
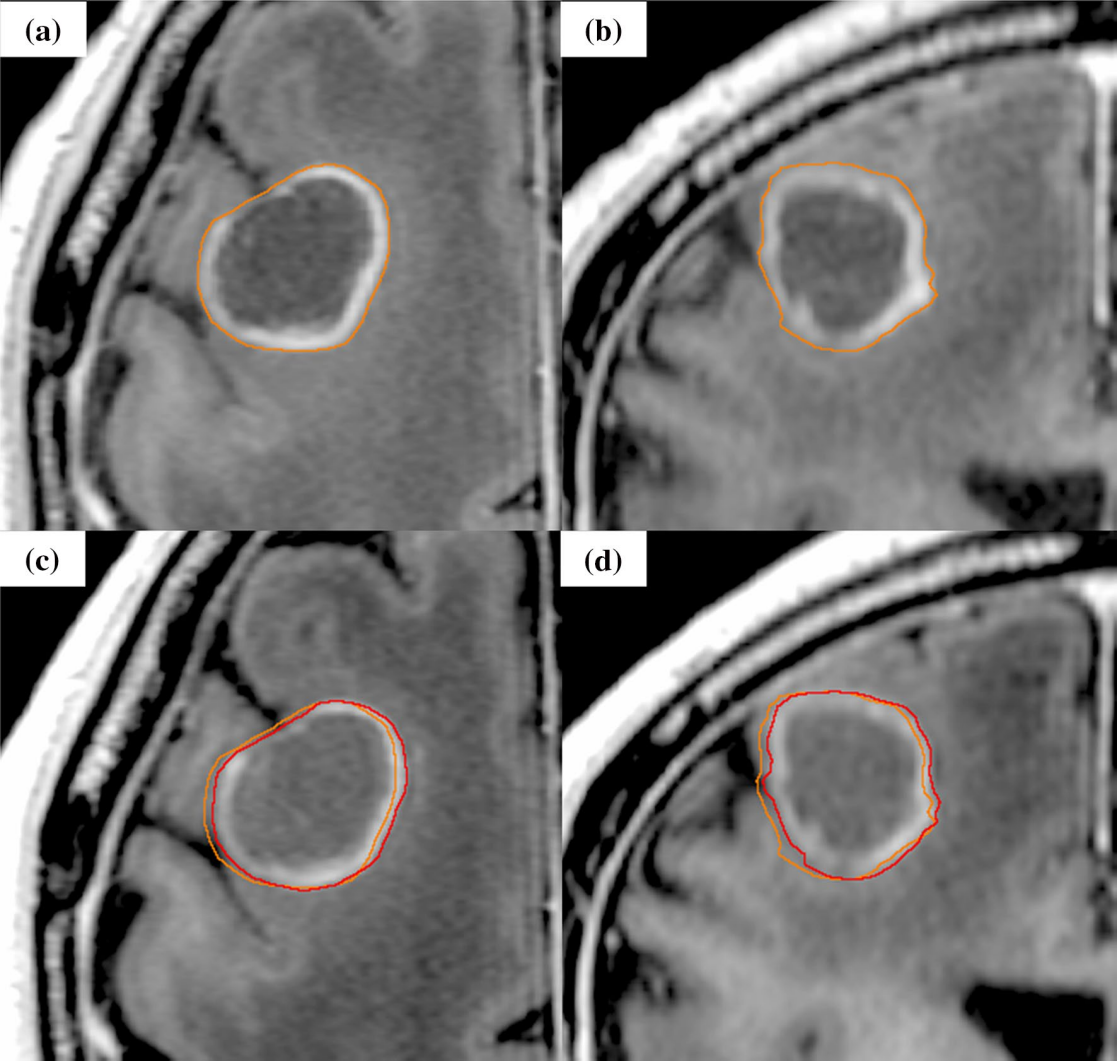
The case of displacement during stereotactic radiotherapy. The case was brain metastasis of which primary tumor was bladder urothelial carcinoma. Orange line shows the gross tumor volume (GTV) of the initial plan, and red line shows GTV at the modification. The tumor shifted to the left.** a, b** Axial, Coronal: T1-weighted magnetic resonance imaging (MRI) with gadolinium (Gd) of the initial plan.** c, d** Axial, Coronal: T1-weighted MRI with Gd before third treatment for the modification (the third day of SRT start)

**Fig. 5:**
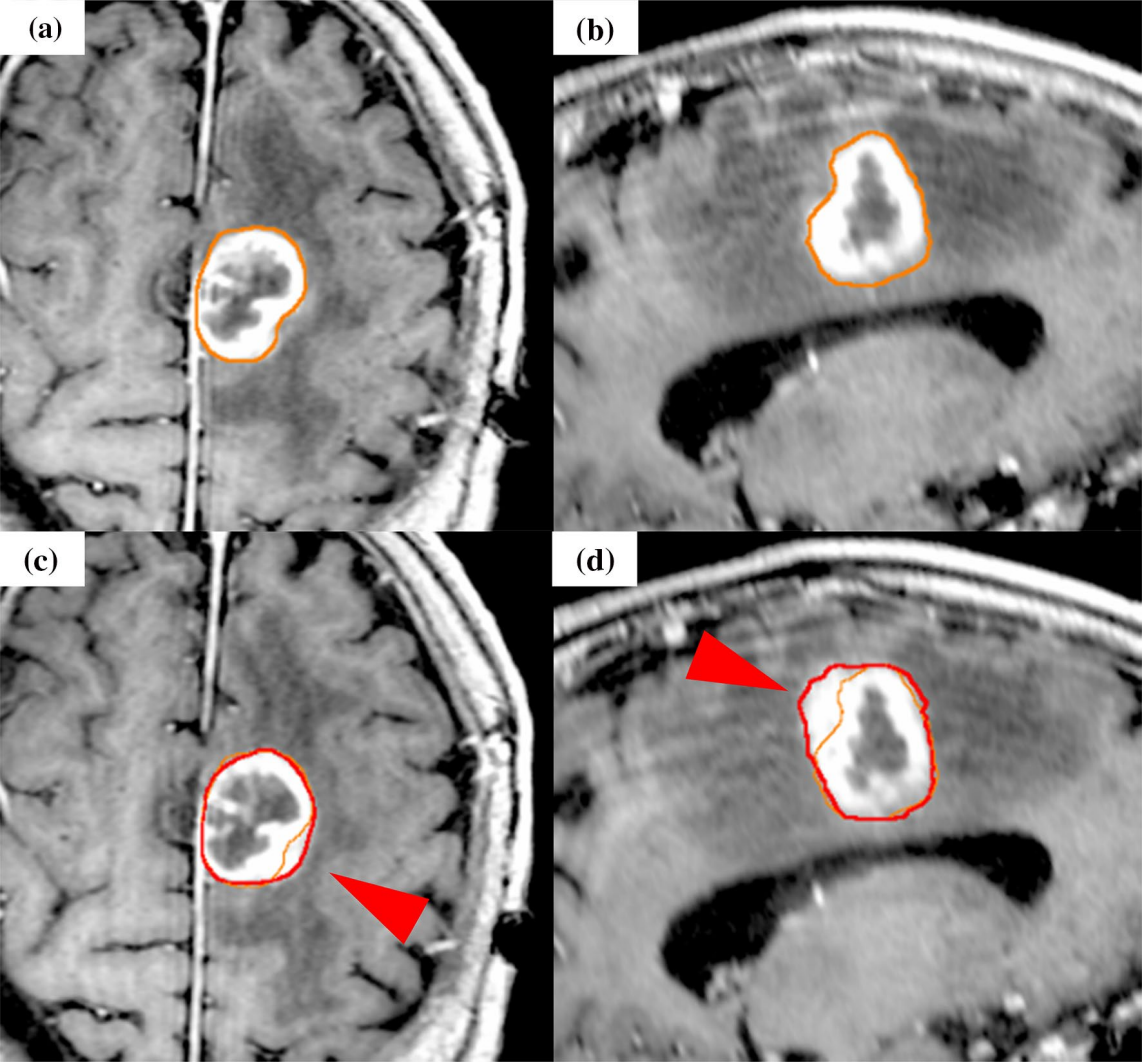
The case of shape change during stereotactic radiotherapy. The case was brain metastasis of which primary tumor was salivary gland pleomorphic adenoma. Orange line shows the gross tumor volume (GTV) of the initial plan, and red line shows GTV at the modification. A slight change of GTV delineation was observed (red arrow).** a, b** Axial, Sagittal: T1- weighted magnetic resonance imaging (MRI) with gadolinium (Gd) of the initial plan.** c, d** Axial, Sagittal: T1-weighted MRI with Gd before third treatment for the modification (the sixth day of SRT start)

Of the 27 large brain metastases, 15 (55.6%) required the treatment plan modification. Table 3 shows the characteristics of lesions with the modification. Of the 15 lesions that required the modification, primary tumor sites were lung in 4, breast in 1, gastrointestinal tract in 3, ovary in 2, skin in 2, and other in 3. Six (40.0% of the lesions required the modification) lesions were previously received intracranial treatment. Median tumor diameter was 2.7 cm (range 2.1–4.0 cm), median GTV size was 9.8 cc (range 3.2–33.0 cc), and median PTV size was 13.0 cc (range 4.8–39.7 cc). The total prescribed doses were 35 Gy in 5 fractions in 10 lesions, and 40 Gy in 8 fractions in 5 lesions. The median GTV D99 with prescribed dose of 35 Gy and 40 Gy were 39.3 Gy (range 36.8–40.3 Gy) and 46.6 Gy (range 44.1–47.4 Gy), respectively. The reasons of the modification were tumor reduction in 6 lesions, tumor enlargement in 3 lesions, displacement in 3 lesions, and shape change in 3 lesions. Figures 2, 3, 4, 5 show the cases with treatment plan modification during the treatment period. The timing of the modification was before second treatment in 1 lesion, third treatment in 2 lesions, fourth treatment in 11 lesions, and seventh treatment in 1 lesion. In the cases with tumor reduction, the GTV and PTV size decreased by 26.2% (range 20.3–43.8%) and 32.7% at mean (range 18.9–41.5%), respectively. With the modification, the brain V70 decreased by 6.5 cc at mean (range 2.3–10.8 cc). In the cases with tumor enlargement, the GTV and PTV size increased by 23.9% (range 10.6–46.4%) and 23.2% at mean (range 12.9–43.0%), respectively. All lesions with tumor enlargement had cystic components on MRI and the pathological status of primary tumor was not adenocarcinoma. The cause of tumor enlargement was enlarged cystic component. In the cases with displacement, the shift of center of PTV ranged from 1.0 mm to 1.7 mm. In the cases with tumor enlargement, displacement, and shape change, the mean new GTV D99 of previous plan and after the plan modification were 33.1 Gy and 38.6 Gy with the prescribed dose of 35 Gy, and those were 42.9 Gy and 45.4 Gy with the prescribed dose of 40 Gy, respectively. The new GTV D99 was significantly improved by the modification (*p* = 0.0287).

**Table 3:** Characteristics of lesions with the treatment plan modification

Tumor number	Primary tumor	Pathological status	Previous treatment	Cyctic lesion	Steroid prior to SRT	Diameter (cm)	GTV (cc)	PTV (cc)	Prescribed dose	GTV D99 of initial treatment (Gy)	Treatment method	Reason of modification	Timing of modification (befre Xth treatment)
5	Breast	Small cell carcinoma	Local radiation therapy	No	No	2.3	6.5	11.9	35Gy/5fr	40.1	VMAT	Tumor reduction	4th
6	Lung	Adenocarcinoma	None	Yes	Yes	2.6	4.7	8.7	35Gy/5fr	40.3	DCAT	Tumor reduction	4th
7	Skin	Melanoma	WBRT	Yes	No	2.8	11.0	14.9	35Gy/5fr	36.8	VMAT	Tumor enlargement	4th
8	Skin	Melanoma	WBRT	Yes	No	3.2	17.1	22.5	35Gy/5fr	37.0	VMAT	Tumor enlargement	4th
10	Lung	Adenocarcinoma	None	Yes	No	3.6	23.6	34.9	40Gy/8fr	46.2	VMAT	Tumor reduction	7th
11	Ovary	Adenocarcinoma	None	No	No	3.5	22.4	31.4	40Gy/8fr	47.1	VMAT	Displacement	4th
12	Ovary	Adenocarcinoma	None	No	No	2.1	5.0	8.3	40Gy/8fr	46.6	VMAT	Displacement	4th
13	Nasal cavity	Small cell carcinoma	SRT	No	No	2.1	4.3	6.7	35Gy/5fr	39.7	VMAT	Shape change	3rd
16	Lung	Not available	Local radiation therapy	Yes	Yes	3.4	20.3	28.7	40Gy/8fr	47.4	VMAT	Tumor reduction	4th
18	Colon	Adenocarcinoma	None	No	No	2.8	11.2	15.9	35Gy/5fr	39.2	VMAT	Tumor reduction	3rd
19	Bladder	Urothelial carcinoma	None	Yes	No	2.4	6.9	10.0	35Gy/5fr	39.7	VMAT	Displacement	4th
20	Esophagus	Small cell carcinoma	WBRT	No	No	2.2	5.3	8.8	35Gy/5fr	39.2	VMAT	Tumor reduction	4th
22	Salivary gland	Pleomorphic adenoma	None	Yes	No	2.7	9.8	13.0	35Gy/5fr	38.8	VMAT	Shape change	4th
25	Lung	Adenocarcinoma	none	No	No	2.1	3.2	4.8	35Gy/5fr	39.4	VMAT	Shape change	2nd
27	Esophagus	Squamous cell carcinoma	None	Yes	Yes	4.0	33.0	39.7	40Gy/8fr	44.1	VMAT	Tumor enlargement	4th

*GTV* gross tumor volume,* PTV* planning target volume,* VMAT* volumetric-modulated arc therapy,* DCAT* dynamic conformal arc therapy,* WBRT* whole brain radiation therapy,* SRT* stereotactic radiotherapy,* GTV D99* dose that covers 99% of the GTV

On the other hand, of the 12 brain metastases that did not require the plan modification, there was little change in the size, shift, and shape of the tumor. Median tumor diameter, median GTV size, and median PTV size based on MRI before and during SRT were 2.6 cm (range 2.1–3.1 cm) and 2.6 cm (range 2.1–3.1 cm), 7.9 cc (range 3.2–15.5 cc) and 7.8 cc (range 3.2–15.4 cc), and 11.0 cc (range 4.9–20.5 cc) and 10.7 cc (range 5.1–20.4 cc), respectively. The change of GTV size ranged from −7.0% to +7.0%. The shift of center of PTV was 0.1 mm (range 0–0.7 mm) at median. The mean GTV D99 based on MRI before and during SRT with prescribed dose of 35 Gy were 38.8 Gy (range 37.5–40.0 Gy) and 38.3 Gy (range 37.5–40.1 Gy), and those with that of 40 Gy were 44.5 Gy (range 44.2–44.7 Gy) and 44.6 Gy (range 44.3–45.0 Gy), respectively. In the cases that did not require the plan modification, there was no significance between GTV D99 and new GTV D99 (*p* = 0.1942).

Table 4 shows the predictive factors of the treatment plan modification. Pathological status (adenocarcinoma vs others) was the predictive factor of tumor enlargement, and displacement and shape change (*p* = 0.0013, and *p* = 0.0450, respectively). In the cases of which the pathological status was adenocarcinoma, the necessity of the treatment plan modification due to tumor enlargement, and displacement and shape change was significantly low. The timing of steroid administration was the predictive factor of tumor reduction, and displacement and shape change (*p* = 0.0358, and *p* = 0.0027, respectively). In the lesions those were administered steroid after the start of SRT, there were significantly more cases required the modification due to tumor reduction, and displacement and shape change.

**Table 4:** Predictive factors of the treatment plan modification

	Tumor reduction	Tumor enlargement	Displacement and shape change
Sex (male vs female)	0.7066	0.6048	1.0000
Pathological status (adenocarcinoma vs others)	0.1186	0.0013	0.0450
Location (frontal lobe vs others)	0.1491	0.4376	0.3173
Exist of cystic component on MRI (yes or no)	0.7066	0.5023	0.8139
Previous treatment (yes or no)	0.7933	0.2918	0.4568
PTV size (≤11.9 cc vs >11.9 cc)	0.9493	0.0701	0.7324
Dose per fraction (5fractions vs 8 fractions)	0.8247	0.7703	0.7098
Timing of steroid administration (prior vs after SRT start)	0.0358	0.1709	0.0027

All are* P* values

*PTV*, planning target volume

## Discussion

This study reported MRI appearance changes during linac-based SRT for large brain metastases, and analyzed the lesions necessitating treatment plan modification. Some studies reported about tumor changes in a short period between planning MRI and STI start, such as tumor size and shift [12, 13]. However, there were few studies mentioned about the detail changes of tumor during SRT periods [9, 10]. In this study, we focused on the change of tumor size, shape, and geometry during SRT, and about half of the total lesions needed the modification during SRT. The PTV size changed up to 43.0%. As SRT needs good target conformity and the PTV margin was small, the tumor size change during treatment period might cause unnecessary irradiation to normal tissue or dose reduction to target lesions. Additionally, even if there was no change of tumor size and shape, the geometrical shift might occur. In this study, the shift of center of PTV was a maximum of 1.7 mm. Indeed, unexpected dose reduction to the tumor was observed in the cases of tumor enlargement, displacement, and shape change. This insufficient target coverage might cause poor local control. Recent studies have reported that tumor changes during SRT resulted in large declines of PTV dose coverage and a conformity index [9, 10]. Hessen et al. mentioned that repeating the MRI during fractionated SRS and registration with the treatment plan provided important insight about the treatment accuracy [10]. Our results also supported their studies. Therefore, the treatment plan evaluation and modification during the treatment period were important.

Next, we refer to the predictive factors of the treatment plan modification. We revealed that pathological status and the timing of steroid administration might be the predictive factors of tumor changes. In the cases of which the pathological status was adenocarcinoma, the necessity of the modification due to tumor changes was significantly low. Adenocarcinoma is traditionally known to have a slow radiation response, and this might be the one reason of the low necessity of the modification. The timing of steroid administration was the predictive factor of tumor reduction, displacement and shape change. The correlation between the shift of center of PTV and change in edema volume was mentioned in previous report [13]. As steroid administration reduced the surrounding edema [14], displacement and shape change might have been caused. In addition, steroid administration was indicated to slightly decrease tumor volume by loss of interstitial fluid in the tumor or restoration of the blood-brain barrier [15]. Therefore, the lesions administrated steroid after SRT start might be more affected than those of which administered steroid prior to SRT. As the radiation necrosis is related to irradiated volume [16], in the cases with tumor reduction, the modification that reduce unnecessary irradiation to normal brain is important. On the other hand, although not a significant factor, it was noted that the all cases with tumor enlargement had cystic component and the cause of tumor enlargement was enlarged cystic component. As the target coverage was decreased in the cases with tumor enlargement, the metastases with cystic component required caution.

As mentioned above, the evaluation of the treatment plan modification was important. In the future, the MRI-linac machine may be one of solutions. However, in many institutions, frequent MRI may be considered difficult, especially in a short duration of SRT. It is important to carefully select cases that need the modification. The most important case is that the tumor is not covered enough by the prescribed dose. Therefore, we thought MRI for the modification during treatment period was necessary in SRT for large brain metastases of which pathological status was not adenocarcinoma and steroid was not administered prior to SRT.

This study had several limitations, including its retrospective and single institution experience, and almost 40% of brain metastases were previously treated. In addition, not all patients underwent MRI during SRT at the same time. If there are two or more days off during the SRT period due to weekend days and holiday, we considered to perform MRI immediately after that. These facts introduced potential biases. However, this study was meaningful in reporting the tumor changes during the short term of SRT and indicating the importance of the treatment plan modification during the treatment period. We reported this research to call everybody’s attention and so that the prescribed dose is properly administered.

In conclusions, this study reported the MRI appearance changes during SRT for large brain metastases. As tumor changes might occur even during the short treatment period of SRT, the treatment plan evaluation and modification during the treatment period were important in SRT with five and more fractions for large brain metastases of which pathological status was not adenocarcinoma and steroid was not administered prior to SRT, in particular.
